# Dermatopathic Lymphadenopathy With Increased IgG4-Positive Plasma Cells: Erratum

**DOI:** 10.1097/01.md.0000467315.91878.6e

**Published:** 2015-06-26

In the article “Dermatopathic Lymphadenopathy With Increased IgG4-Positive Plasma Cells”,^1^ which appeared in Volume 94, Issue 22 of *Medicine*, Table 1 was formatted incorrectly. The article has since been corrected online.
